# Diastolic index as a short-term prognostic factor in heart failure with preserved ejection fraction

**DOI:** 10.1136/openhrt-2020-001469

**Published:** 2020-12-17

**Authors:** Shiro Hoshida, Shungo Hikoso, Yukinori Shinoda, Koichi Tachibana, Tomoko Minamisaka, Shunsuke Tamaki, Masamichi Yano, Takaharu Hayashi, Akito Nakagawa, Yusuke Nakagawa, Takahisa Yamada, Yoshio Yasumura, Daisaku Nakatani, Yasushi Sakata, Osaka CardioVascular Conference Investigators, Yasushi Sakata

**Affiliations:** 1 Department of Cardiovascular Medicine, Yao Municipal Hospital, Yao, Osaka, Japan; 2 Department of Cardiovascular Medicine, Osaka University Graduate School of Medicine, Suita, Osaka, Japan; 3 Division of Cardiology, Osaka General Medical Center, Osaka, Japan; 4 Division of Cardiology, Osaka Rosai Hospital, Sakai, Osaka, Japan; 5 Cardiovascular Division, Osaka Police Hospital, Osaka, Japan; 6 Division of Cardiovascular Medicine, Amagasaki Chuo Hospital, Amagasaki, Hyogo, Japan; 7 Department of Medical Informatics, Osaka University Graduate School of Medicine, Suita, Osaka, Japan; 8 Division of Cardiology, Kawanishi City Hospital, Kawanishi, Hyogo, Japan

**Keywords:** echocardiography, heart failure, diastolic, biomarkers

## Abstract

**Objective:**

During follow-up time, the value of prognostic factors may change, especially in the elderly patients, and the altered extent may affect the prognosis. We aimed to clarify the significance of the ratio of diastolic elastance (Ed) to arterial elastance (Ea), (Ed/Ea=(E/e’)/(0.9×systolic blood pressure)), an afterload-integrated diastolic index, in relation to follow-up periods and other laboratory factors, on the prognosis of elderly patients with heart failure with preserved ejection fraction (HFpEF).

**Methods:**

We studied 552 HFpEF patients hospitalised for acute decompensated heart failure (men/women: 255/297). Blood testing and transthoracic echocardiography were performed before discharge. The primary endpoint was all-cause mortality.

**Results:**

During a median follow-up of 508 days, 88 patients (men/women: 39/49) had all-cause mortality. During the first year after discharge, Ed/Ea (p=0.045) was an independent prognostic factor in association with albumin (p<0.001) and N-terminal pro-brain natriuretic peptide (NT-proBNP, p=0.005) levels after adjusting for age and sex in the multivariate Cox hazard analysis. However, at 1 to 3 years after discharge, no other significant prognostic factors, except for albumin level (p=0.046), were detected. In the subgroup analysis, albumin, but not NT-proBNP level, showed a significant interaction with Ed/Ea for prognosis (p=0.047).

**Conclusion:**

The prognostic significance of a haemodynamic parameter such as Ed/Ea may be valid only during a short-term period, but that of albumin was persisting during the entire follow-up period in the elderly patients. The clinical significance of prognostic factors in HFpEF patients may differ according to the follow-up period.

**Key questions**

**What is already known about this subject?**

- Diastolic function cannot be optimally assessed by one measure alone, but is best assessed using a combination of several echocardiographic indices in patients with heart failure with preserved ejection fraction (HFpEF). We previously reported the ratio of left ventricular (LV) diastolic elastance (Ed) to arterial elastance (Ea) as a novel index of afterload-integrated diastolic function, which can be calculated as (E/e’)/(0.9×systolic blood pressure).

**What does this study add?**

- The prognostic significance of a haemodynamic parameter such as Ed/Ea may be valid only during a short-term period, but that of albumin is persisting during the entire follow-up period in the elderly patients. Ed/Ea showed a significant interaction with albumin level for prognosis. The clinical significance of prognostic factors in HFpEF patients may differ according to the follow-up period.

**How might this impact on clinical practice?**

- Haemodynamic parameters regarding LV diastolic function could change rigorously after discharge of the indexed admission for heart failure. A large-scale, prospective study is needed to clarify the differences in prognostic factors in relation to follow-up period, in addition to the changes in haemodynamic parameters, including Ed/Ea, among patients with HFpEF.

## Introduction

Diastolic function cannot be optimally assessed by one measure alone, but is best assessed using a combination of several echocardiographic indices in patients with heart failure with preserved ejection fraction (HFpEF).^1–3^ Patients with HFpEF have an increased left atrial volume (LAV), which is an index of LAV overload, and an increased E/e’, which is an index of left atrial (LA) pressure overload.^4 5^ We previously reported the ratio of left ventricular (LV) diastolic elastance (Ed) to arterial elastance (Ea) as a novel index of afterload-integrated diastolic function, which can be calculated as (E/e’)/(0.9×systolic blood pressure).^6^ Ed/Ea exhibits an LA pressure relative to the systemic pressure.^7^ Thus, the Ed/Ea ratio may reflect the left-sided heart function, including the atrio-ventricular-arterial interaction, under a preserved LV ejection fraction (LVEF).

We recently reported that Ed/Ea may be a useful independent determinant of all-cause mortality in the elderly patients with HFpEF showing sinus rhythm.^8^ However, during follow-up time, the value of prognostic factors may change, especially in the elderly patients, and the altered extent may affect the prognosis. LVEF is reported to change after discharge in patients with heart failure.^9 10^ The changes in the N-terminal pro-brain natriuretic peptide (NT-proBNP) levels after discharge have been related to prognostic changes in patients with HFpEF.^11^ Furthermore, for the clarification of prognostic factors for HFpEF, we typically focus on combining echocardiographic markers with other types of general predictive factors. This study aimed to clarify the differences in the role of Ed/Ea in relation to follow-up time and other common prognostic factors for predicting all-cause mortality in patients with HFpEF.

## Methods

### Study subjects

Of the 637 patients with prognostic data recruited from the Prospective Multicenter Observational Study of Patients with Heart Failure with Preserved Ejection Fraction (PURSUIT HFpEF) registry (2016.6 to 2019.4), we excluded 85 with poor echocardiographic data. Therefore, we enrolled 552 patients (LVEF ≥50%; men/women, 255/297; mean age, 81 years) at discharge during the index hospitalisation for acute decompensated heart failure (ADHF). The PURSUIT HFpEF registry is a prospective multicentre observational registry in which collaborating hospitals in the Osaka region of Japan record the clinical, echocardiographic and outcome data of patients with HFpEF (UMIN-CTR ID: UMIN000021831).^5^

### Echocardiography and laboratory testing

Transthoracic echocardiography was performed when patients were in a stable condition before discharge. Echocardiographic measurements were obtained according to the American Society of Echocardiography (ASE) or European Society of Echocardiography guidelines.^2 12^ Volumetry was standardised using the modified Simpson’s rule. As a marker of LA pressure overload for estimating LV diastolic function, we examined afterload-integrated Ed/Ea ((E/e’)/(0.9×systolic blood pressure)).^5 6 13^ Systolic blood pressure was examined during echocardiographic measurements. As the relative markers of LAV overload, we evaluated LAV index (LAVI) and the ratio of stroke volume (SV) to LAV.^7^ Moreover, serum NT-proBNP and albumin levels, haemoglobin concentration and estimated glomerular filtration rate (eGFR) were examined when patients were stable before discharge.

### Follow-up/clinical outcome

All patients were followed up at each hospital after discharge. Survival data were obtained by dedicated coordinators and investigators through direct contact with patients and their physicians at the hospital, in an outpatient setting, via telephone interview with their families or by mail. The primary endpoint of this study was all-cause mortality.

### Patient and public Involvement

The PURSUIT HFpEF registry is managed in accordance with the principles of the Declaration of Helsinki. All participants provided written informed consent regarding the design and conduct of the study during the indexed hospitalisation. We performed only essential examinations in routine clinical practice.

### Statistical analysis

Continuous variables are expressed as means±SD, whereas categorical variables are presented as frequencies and percentages. Differences in categorical variables between the groups were assessed using the χ^2^ tests, while those in continuous variables were assessed using the Student’s or Welch’s t-tests, as appropriate. Correlations were assessed using the Pearson or Spearman coefficients, and p values were examined using regression analysis. Cut-off points of the prognostic factors for all-cause mortality were evaluated using a receiver operating characteristic (ROC) curve analysis. Survival curves were estimated using the Kaplan-Meier survival analysis, and the groups were compared using the log-rank test. Landmark analysis was performed 1 year after discharge. The Cox proportional hazards regression analysis was initially evaluated in a univariate analysis. Subsequently, a multivariate Cox proportional hazards regression analysis was conducted with the echocardiographic and laboratory data, adjusting for age, sex and significant variables of a univariate analysis. In the subgroup analysis, the effect of Ed/Ea on prognosis was evaluated during the first year after discharge in a Cox regression analysis. The interaction was also examined between Ed/Ea and each variable. P values <0.05 were considered statistically significant. All statistical analyses were performed using the EZR (Saitama Medical Center, Jichi Medical University, Saitama, Japan), a graphical user interface for R (The R Foundation for Statistical Computing, Vienna, Austria).

## Results

### Clinical and laboratory characteristics of patients with HFpEF

During a median follow-up of 508 days, 88 patients (men/women: 39/49) had all-cause mortality. We observed significant differences between patients with and without all-cause mortality in terms of age (p<0.001), albumin (p<0.001), haemoglobin (p<0.001) and NT-proBNP (p=0.004) levels (online supplemental table 1). We observed no significant differences in medications or the incidence of male sex, atrial fibrillation, hypertension, diabetes mellitus, dyslipidaemia and coronary artery disease between the two groups. Regarding echocardiographic parameters, E/e’ (p<0.001) and Ed/Ea (p<0.001), but not LAVI, SV/LAV, LVEF or tricuspid annular plane systolic excursion (TAPSE) at discharge, significantly differed between patients with and without all-cause mortality (online supplemental table 1). Although the data are not shown, the deceleration time of the E wave, septal e’, lateral e’ and E/A did not significantly differ between the groups.

10.1136/openhrt-2020-001469.supp1 Supplementary data

The NT-proBNP log-transformed level was modestly correlated with echocardiographic indices, such as LAVI (r=0.248, p<0.001), SV/LAV (r=−0.216, p<0.001) and Ed/Ea (r=0.171, p<0.001). Evaluation of the correlations between the indices of LA pressure and volume overload showed that Ed/Ea was modestly correlated with LAVI (r=0.153, p<0.001) or SV/LAV (r=−0.123, p=0.006).

### Prognostic analysis

**Figure 1:**
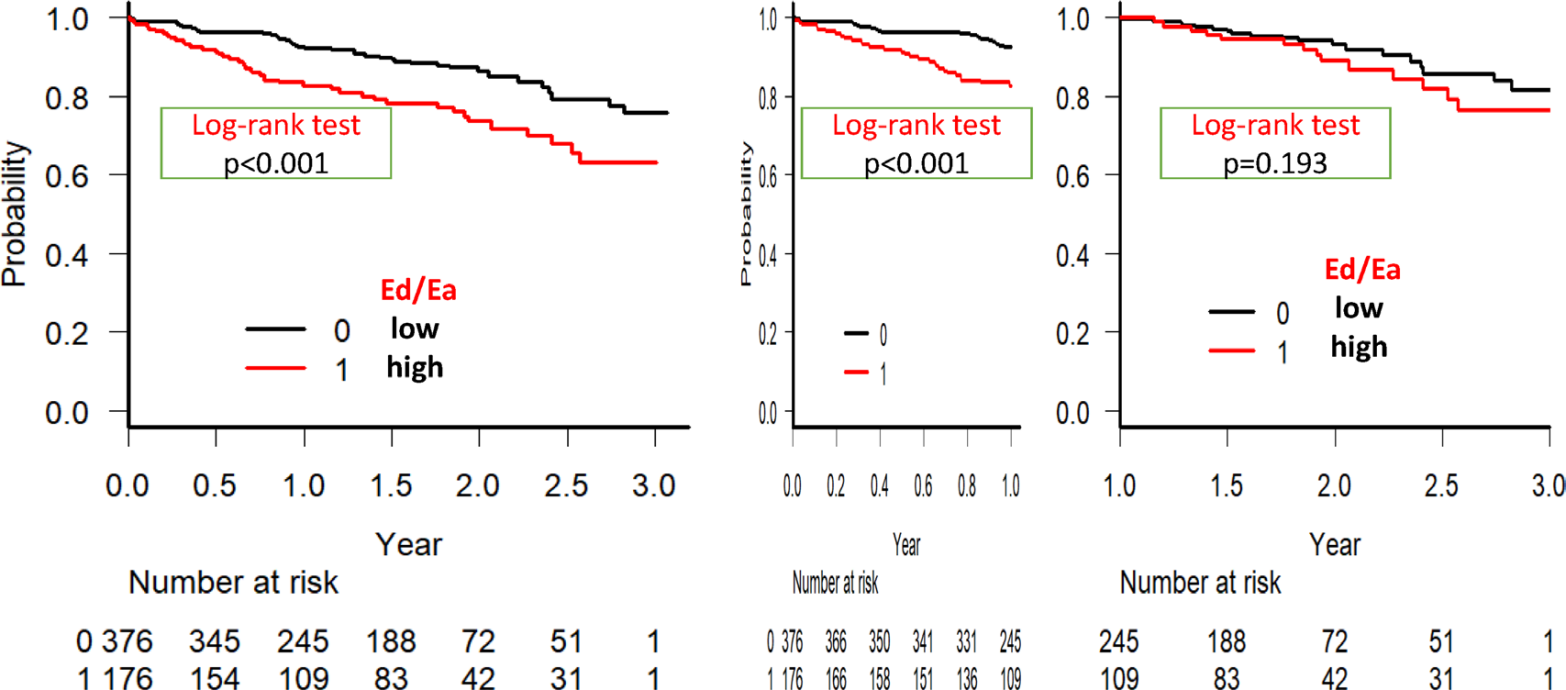
The ratio of diastolic elastance (Ed)/arterial elastance (Ea) as a prognostic factor in the Kaplan-Meier survival curve analysis of patients with heart failure with preserved ejection fraction: differences are observed according to the follow-up time by landmark analysis. Ed/Ea >0.144 is a significant prognostic factor for all-cause mortality during the entire follow-up time and up to 1 year after, but not 1 to 3 years after discharge.

**Figure 2:**
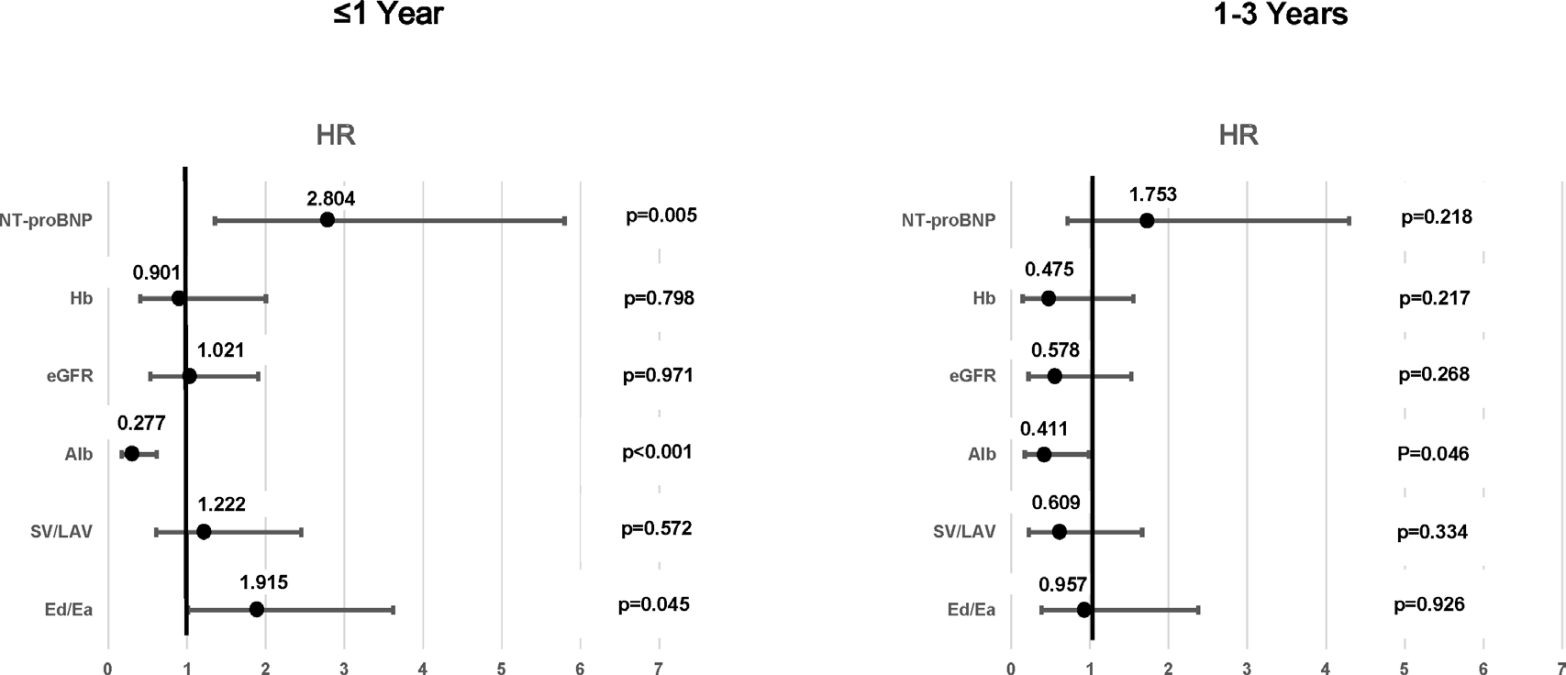
Multivariate Cox hazard analysis in the echocardiographic and laboratory data adjusting with age, sex and variables in this figure during different follow-up time (≤1 year and 1 to 3 years) in patients with heart failure with preserved ejection fraction. HR and 95% CI for each variable are shown. Alb, albumin; Ea, arterial elastance; Ed, diastolic elastance; eGFR, estimated glomerular filtration rate; Hb, haemoglobin; LAV, left atrial volume; NT-proBNP, N-terminal pro-brain natriuretic peptide; SV, stroke volume.

The areas under the curve and cut-off points of each parameter were evaluated in the ROC curve analysis for the prediction of all-cause mortality. Table 1 shows the comparison with clinical characteristics between patients with lower and higher Ed/Ea than the cut-off point by the ROC curve analysis. Age, blood pressure, eGFR, NT-proBNP levels, male sex and LAVI were significantly different between these two groups. The Kaplan-Meier survival curve analysis (online supplemental figure 1; figure 1) and a univariate Cox hazard analysis (table 2) revealed that Ed/Ea and SV/LAV, but not LAVI, in case of echocardiographic data, and albumin, haemoglobin, NT-proBNP levels and eGFR, in case of laboratory data, were significant as prognostic factors. During the first year after discharge, the independent prognostic factors were Ed/Ea, and albumin and NT-proBNP levels in the multivariate Cox hazard analysis (figure 2). At 1 to 3 years after discharge, however, no independently significant prognostic factors, except for albumin level, were detected (figure 2). When we performed landmark analysis in the Kaplan-Meier survival curve analysis, differences were observed according to the follow-up time. Ed/Ea >0.144 was a significant prognostic factor for all-cause mortality during whole follow-up time and up to 1 year after, but not 1 to 3 years after discharge (figure 1). Although not shown, albumin and NT-proBNP levels, but not Ed/Ea, SV/LAV, eGFR or haemoglobin, were independently significant in prognosis after adjusting for age and sex during the whole follow-up period in the multivariate Cox regression analysis.

**Table 1:** Clinical characteristics before discharge in patients with low and high Ed/Ea

	Ed/Ea		P value (low vs high)
	≤0.144 (n=376)	>0.144 (n=176)	
Age, years	80±9	83±9	0.002
Male sex, n (%)	195 (52)	60 (34)	<0.001
Systolic blood pressure, mm Hg	122±17	116±16	<0.001
Diastolic blood pressure, mm Hg	67±11	63±11	<0.001
Heart rate, bpm	72±13	71±14	0.649
Atrial fibrillation, n (%)	142 (38)	58 (33)	0.273
Chronic obstructive pulmonary disease, n (%)	26 (7)	9 (5)	0.534
Coronary artery disease, n (%)	72 (19)	46 (26)	0.062
Diabetes mellitus, n (%)	130 (35)	64 (36)	0.681
Dyslipidaemia, n (%)	156 (41)	78 (44)	0.531
Hypertension, n (%)	320 (85)	157 (89)	0.190
Laboratory data			
Albumin, g/dL	3.4±0.5	3.4±0.5	0.607
eGFR, mL/min/1.73 m^2^	44.3±18.9	39.2±18.7	0.003
Haemoglobin, g/dL	11.5±2.1	11.1±1.9	0.061
N-terminal pro-brain natriuretic peptide, pg/mL	2202±4406	3669±8963	0.014
Echocardiographic data			
LAD, mm	43±8	46±8	<0.001
LAVI, mL/m^2^	52±24	60±26	<0.001
LVEDVI, mL/m^2^	54±21	54±20	0.989
LVESVI, mL/m^2^	22±11	21±10	0.405
SVI, mL/m^2^	32±12	33±13	0.515
SV/LAV	0.74±0.41	0.65±0.35	0.010
LVEF, %	60±7	61±8	0.311
TAPSE, mm	17.7±4.4	17.4±4.6	0.481
E/e'	10.9±2.9	19.6±4.8	<0.001
Medications			
Beta-blockers, n (%)	207 (55)	96 (55)	0.911
Calcium channel blockers, n (%)	197 (52)	93 (53)	0.921
Diuretics, n (%)	309 (82)	152 (86)	0.217
RAAS inhibitors, n (%)	272 (72)	136 (77)	0.218
Statins, n (%)	122 (32)	65 (37)	0.299

Values are presented as means±SD or numbers (%).

Ea, arterial elastance; Ed, diastolic elastance; eGFR, estimated glomerular filtration rate; LAD, left atrial diameter; LAV, left atrial volume; LAVI, left atrial volume index; LVEDVI, left ventricular end-diastolic volume index; LVEF, left ventricular ejection fraction; LVESVI, left ventricular end-systolic volume index; RAAS, renin-angiotensin-aldosterone system; SV, stroke volume; SVI, stroke volume index; TAPSE, tricuspid annular plane systolic excursion.

**Table 2:** Survival analysis in patients with heart failure with preserved ejection fraction

	ROC curve		Kaplan-Meier	Cox hazard analysis	
	Cut-off	AUC	P value	Univariate	
				P value	Ratio (95% CI)
Ed/Ea	0.144	0.623	<0.001	<0.001	2.056 (1.353 to 3.124)
SV/LAV	0.487	0.546	0.022	0.024	0.592 (0.376 to 0.933)
Alb	3.2	0.676	<0.001	<0.001	0.293 (0.191 to 0.451)
eGFR	40	0.566	0.020	0.021	0.604 (0.393 to 0.927)
Hb	12.3	0.613	0.003	0.004	0.433 (0.244 to 0.766)
NT-proBNP	1220	0.697	<0.001	<0.001	3.837 (2.333 to 6.311)

Alb, albumin; AUC, area under the curve; Ea, arterial elastance; Ed, diastolic elastance; eGFR, estimated glomerular filtration rate; Hb, haemoglobin; LAV, left atrial volume; NT-proBNP, N-terminal pro-brain natriuretic peptide; ROC, receiver operating characteristic; SV, stroke volume.

In the subgroup analysis during the first year after discharge, Ed/Ea was a significant prognostic factor in patients with higher albumin or lower eGFR levels in a Cox regression analysis, although the interaction was significant only between Ed/Ea and albumin (table 3). In patients with higher NT-proBNP, Ed/Ea was a significant prognostic factor, but no significant interaction was observed between Ed/Ea and NT-proBNP level (table 3). Irrespective of the haemoglobin level, Ed/Ea was significant for prognosis in patients with HFpEF.

**Table 3:** Subgroup analysis: Ed/Ea as a prognostic factor in patients with heart failure

		Cox hazard analysis		Interaction
		P value	Ratio (95% CI)	P value
Alb	low	0.346	1.405 (0.692 to 2.851)	0.047
	high	<0.001	4.336 (1.819 to 10.34)	
eGFR	low	0.004	2.979 (1.407 to 6.309)	0.26
	high	0.273	1.587 (0.694 to 3.626)	
Hb	low	0.009	2.161 (1.205 to 3.878)	0.353
	high	0.026	4.186 (1.181 to 14.84)	
NT-proBNP	low	0.219	2.052 (0.651 to 6.465)	0.997
	high	0.024	2.038 (1.095 to 3.792)	

Alb, albumin; eGFR, estimated glomerular filtration rate; Hb, hemoglobin; NT-proBNP, N-terminal pro-brain natriuretic peptide.

## Discussion

The prognostic risk factors differed according to the follow-up time in the elderly HFpEF patients. The afterload-integrated diastolic index, Ed/Ea, provided significant prognostic information for predicting all-cause mortality during the first year follow-up, but not thereafter. In the subgroup analysis, although Ed/Ea was useful for predicting prognosis in patients with higher albumin, lower eGFR or higher NT-proBNP, only Ed/Ea and albumin showed a significant interaction for prognosis.

### Prognostic factors in relation to follow-up duration

Blood laboratory data, such as albumin and NT-proBNP levels, were useful for predicting prognosis in HFpEF patients at the follow-up duration of 3 years. To determine the difference in prognostic factors in relation to the follow-up duration, we examined the survival data by a multivariate Cox hazard analysis using two different time points: first year after the enrollment and 1 to 3 years thereafter. During the first year after discharge, Ed/Ea, albumin and NT-proBNP levels were the significant prognostic factors (figure 2). Nevertheless, at 1 to 3 years after the enrolment, there were no significant factors for prognosis except for albumin level. Changes in natriuretic peptide levels have shown prognostic value in patients with HFpEF;^11^ an increase in these levels over 6 months after the study enrolment was associated with an increased prognosis, while a decrease in these levels was associated with outcome improvement. HFpEF does not transition to other conditions, such as HF with reduced LVEF or with mid-range LVEF, especially within 1 year in patients with relatively younger age (mean, 71.7 years) and preserved eGFR (mean, 58.7 mL/min/1.73 m^2^).^9^ However, in elderly patients like our subjects, pathophysiological haemodynamic change may rigorously occur during 1 year after discharge, possibly leading to different haemodynamic conditions that could not be estimated during the enrolment. Since albumin level was independently significant for prognosis during 1 to 3 years after discharge, this common prognostic factor may be important even during the middle-term or long-term follow-up in the elderly patients. In the evaluation of prognostic factors in the elderly patients with HFpEF, one must consider the follow-up duration.

### Subgroup analysis

Single measures of risk are rarely sufficient for the accurate estimation of prognosis in complex diseases, such as HFpEF.^14^ Among the general prognostic factors, the effect of higher Ed/Ea on prognosis was different in patients with laboratory prognostic factors. In patients with a higher albumin or lower eGFR level, the effect of higher Ed/Ea on prognosis was prominent, although the interaction was significant only between Ed/Ea and albumin level. In those with lower albumin level (<3.2 g/dL), their cause of death may be accompanied by general conditions related to age-associated factors, such as poor alimentary state, resulting in no more significant effect of haemodynamic condition on prognosis. In contrast, renal dysfunction shows an additive effect on cardiac dysfunction for prognosis, leading to the associated prognostic effect of higher Ed/Ea in patients with lower eGFR. The cause of death in these patients may be related to the worsened haemodynamic state. Irrespective of haemoglobin levels, those with higher Ed/Ea showed poor prognosis. Since no interaction was observed between Ed/Ea and haemoglobin level, the extent of anaemic level did not affect prognosis in the higher Ed/Ea condition. Although anaemia may cause high-output cardiac failure, this type of failure would not relate to prognosis in the elderly patients with HFpEF.

In contrast, the prognostic value of NT-proBNP level has been well established for patients hospitalised for ADHF.^15 16^ Notably, a higher NT-proBNP level in association with higher Ed/Ea closely relates to the poor prognosis in our study. The synergistic effect of these factors was prominent, although no significant interaction was observed between these factors. Since the correlation coefficient was slightly higher between NT-proBNP and an index of LAV overload than that between NT-proBNP and an index of LA pressure overload, the NT-proBNP value may represent the haemodynamic condition reflecting more volume overload in patients with HFpEF. How Ed/Ea and NT-proBNP are related to each other in the cardiac performance deterioration remains to be elucidated. Those with lower NT-proBNP levels may meet their end regardless of their haemodynamic conditions. In fact, the possibility that comorbidities contribute relatively more to prognosis, leading to non-cardiovascular outcomes, is reported in patients with HFpEF with lower NT-proBNP levels.^17^ This issue is in accordance to the results of our study.

### Limitations

We examined all-cause mortality rather than cardiac death because the precise determination of cardiac death was challenging in the elderly patients. Mortality rate was lower than that in other reports for ADHF patients in Japan, such as the Kyoto Congestive Heart Failure registry,^18^ although the mean age of HFpEF patients in that registry was nearly same to our registry. This relatively low mortality rate may affect our results regarding the prognostic significance of Ed/Ea. In this sense, the impact of Ed/Ea on prognosis may be clearer when we examine the composite endpoints composed of all-cause mortality and heart failure hospitalisation. E’ is sometimes very low which affect E/e’ and Ed/Ea in elderly patients with HFpEF.

## Conclusion

Follow-up duration was important in determining Ed/Ea as a prognostic factor in the elderly patients with HFpEF. The pathophysiological haemodynamic state may rigorously change during the first year after discharge, possibly leading to different haemodynamic conditions thereafter. Ed/Ea showed a significant interaction with albumin level for prognosis.
