# Supplementary material for: Diastolic index as a short-term prognostic factor in heart failure with preserved ejection fraction
Source: Open Heart. 2020 Dec 17;7(2):e001469. doi: 10.1136/openhrt-2020-001469 (PMC7747540; doi:10.1136/openhrt-2020-001469)
Supplement: Supplementary data [file openhrt-2020-001469supp001.pdf]

SUPPLEMENTAL MATERIAL

Supplemental Table

Suppl. Table 1. Clinical characteristics before discharge in patients with and without all-cause mortality

	All-cause mortality		***P value***
	**- (n = 464)**	**+ (n = 88)**	***(- vs. +)***
**Age, years**	**80 ± 9**	**85 ± 7**	***<0.001***
**Male sex, n (%)**	**216 (47)**	**39 (44)**	***0.700***
**Systolic blood pressure, mmHg**	**120 ± 17**	**120 ± 19**	***0.896***
**Diastolic blood pressure, mmHg**	**66 ± 11**	**65 ± 11**	***0.731***
**Heart rate, bpm**	**71 ± 13**	**74 ± 14**	***0.085***
**Atrial fibrillation, n (%)**	**166 (36)**	**34 (39)**	***0.608***
**Chronic obstructive pulmonary disease, n (%)**	**27 (6)**	**8 (9)**	***0.248***
**Coronary artery disease, n (%)**	**96 (21)**	**22 (25)**	***0.365***
**Diabetes mellitus, n (%)**	**165 (36)**	**29 (33)**	***0.638***
**Dyslipidemia, n (%)**	**203 (44)**	**31 (35)**	***0.138***
**Hypertension, n (%)**	**404 (87)**	**74 (84)**	***0.452***
**Laboratory data**			
**Albumin, g/dL**	**3.4 ± 0.4**	**3.1 ± 0.5**	***<0.001***
**eGFR, mL/min/1.73 m^2**	**43.1 ± 18.6**	**40.3 ± 21.0**	***0.210***
**Hemoglobin, g/dL**	**11.5 ± 2.0**	**10.7 ± 1.8**	***<0.001***
**N-terminal pro-brain natriuretic peptide, pg/mL**	**2309 ± 4834**	**4479 ± 10758**	***0.004***
**Echocardiographic data**			
**LAD, mm**	**44 ± 8**	**44 ± 8**	***0.808***
**LAVI, mL/m^2**	**53 ± 24**	**59 ± 29**	***0.075***
**LVEDVI, mL/m^2**	**54 ± 20**	**56 ± 22**	***0.401***
**LVESVI, mL/m^2**	**21 ± 11**	**23 ± 13**	***0.262***
**SVI, mL/m^2**	**32 ± 12**	**33 ± 12**	***0.702***
**SV/LAV**	**0.72 ± 0.37**	**0.69 ± 0.48**	***0.608***
**LVEF, %**	**61 ± 8**	**60 ± 8**	***0.319***
**TAPSE, mm**	**17.8 ± 4.4**	**16.8 ± 4.7**	***0.071***
**E/e'**	**13.4 ± 5.3**	**15.6 ± 5.9**	***<0.001***
**Ed/Ea**	**0.125 ± 0.050**	**0.147 ± 0.057**	***<0.001***
**Medications**			

**Beta-blockers, n (%)**	**252 (54)**	**51 (58)**	***0.528***
**Calcium-channel blockers, n (%)**	**242 (52)**	**48 (55)**	***0.680***
**Diuretics, n (%)**	**385 (83)**	**76 (86)**	***0.432***
**RAAS inhibitors, n (%)**	**344 (74)**	**64 (73)**	***0.782***
**Statins, n (%)**	**158 (34)**	**29 (33)**	***0.842***

Values are presented as means ± standard deviations or numbers (%).
eGFR, estimated glomerular filtration rate; LAD, left atrial diameter;
LAVI, left atrial volume index; LVEDVI, left ventricular end-diastolic volume index;
LVESVI, left ventricular end-systolic volume index; SVI, stroke volume index;
SV, stroke volume; LAV, left atrial volume; LVEF, left ventricular ejection fraction;
TAPSE, tricuspid annular plane systolic excursion;
Ed, diastolic elastance; Ea, arterial elastance;
RAAS, renin-angiotensin-aldosterone system

Supplemental Figure

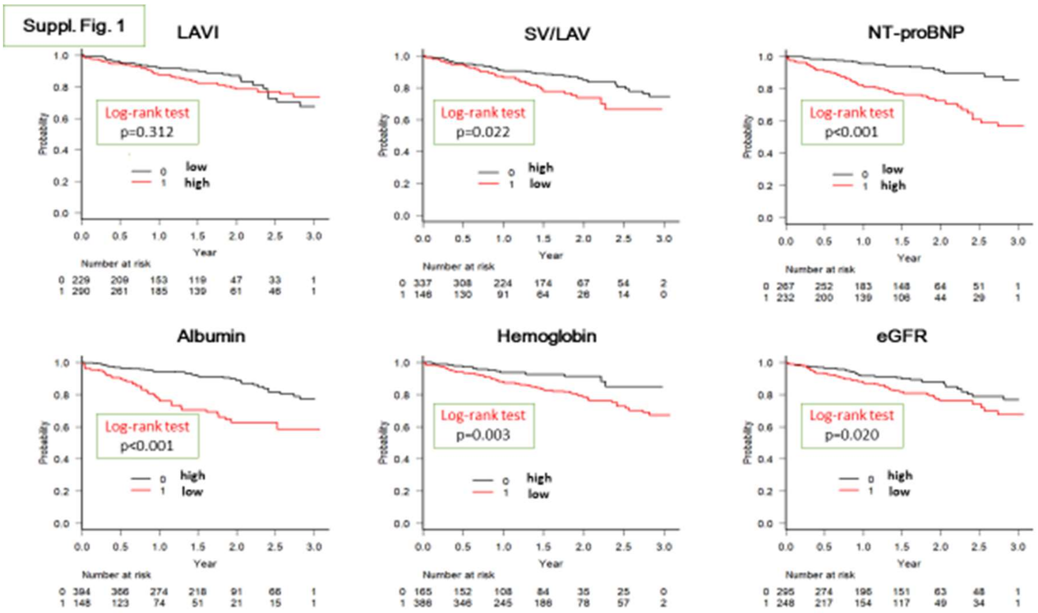

Suppl. Figure 1. Kaplan–Meier survival curve analysis of patients with heart failure with preserved ejection fraction shows that the ratio of stroke volume (SV) to left atrial volume (LAV), N-terminal pro-brain natriuretic peptide (NT-proBNP), albumin, hemoglobin, and estimated glomerular filtration rate (eGFR), but not left atrial volume index (LAVI), are significant prognostic factors for all-cause mortality during the whole follow-up time.
